# Assessment of Airborne Bacterial and Fungal Communities in Shahrekord Hospitals

**DOI:** 10.1155/2021/8864051

**Published:** 2021-04-24

**Authors:** Davood Jalili, MohamadHadi Dehghani, Abdolmajid Fadaei, Mahmood Alimohammadi

**Affiliations:** ^1^Students' Research Committee, Research Center, Tehran University of Medical Sciences, Tehran, Iran; ^2^Department of Environmental Health Engineering, School of Health, Tehran University of Medical Sciences, Tehran, Iran; ^3^Department of Environmental Health Engineering, School of Health, Shahrekord University of Medical Sciences, Shahrekord, Iran

## Abstract

This paper presents information about airborne microorganisms (bacteria and fungi) in the indoor air of two hospitals (Kashani and Hajar) in the city of Shahrekord, Iran. The settle plate technique using open Petri dishes containing different culture media was employed to collect a sample and using Quick Take 30 Sample Pump three days per week for a period of 8 weeks. Standard microbiological methods were employed for the identification of bacterial and fungal isolates. The results showed that the concentration of bacteria in the study area ranged from 0 to 70 cfu/plate/h, while the concentration of fungi was 0 to 280 cfu/plate/h. Also, 12 bacterial and 3 fungal species were isolated and identified with varying frequencies of occurrence, including *Staphylococcus* spp.*, Escherichia coli, Salmonella, Enterobacter, Pseudomonas, Serratia Citrobacter, Proteus,* and *Klebsiella*, while the fungal genera isolated included Yeast, *Aspergillus flavus, and Penicillium*. While the bacterial isolates *Staphylococcus aureus* (20.50%) and *Pseudomonas* (9.10%) were the most predominant airborne bacteria, yeast (22.70%) and *Penicillium* (20.50%) were the most frequently isolated fungal species. The population of microorganisms was the highest during the afternoon. The statistical analysis showed a significant difference between the microbial loads of the two hospitals at *P* < 0.05. The generated data underline the usefulness of monitoring the air quality of the indoor hospital.

## 1. Introduction

Indoor air contains a complex mixture of microorganism species, and intermediate products, such as yeast fungi, molds, bacteria, viruses, and volatile microbial organic compounds.

Exposure to these microbials, fragments of plant tissues, and metabolites may result in life health issues [1, 2]. Airborne bacteria can be toxic, allergenic, and/or infectious [3]. The type of microorganisms and number of organisms present in the air are based on physicochemical factors like temperature, number of occupants, physical quality of the building, humidity, lighting, colloidal suspension, and organic material and food availability. Human activities are also an important determining factor for the diversity of microbes in an area [4]. The presence of airborne microorganisms is the most important subject in indoor environments, such as residential homes, schools, universities hospitals, and care centers [5]. *Staphylococcus aureus*, *Sphingomonas paucimobilis*, *Pseudomonas aeruginosa*, *Stenotrophomonas maltophilia*, and *Clostridium difficile* are common nosocomial bacterial species in hospitals. Fungal infection includes *Aspergillus, Mucor,* and *Rhizopus* [6]. The density of microorganisms in the air can be measured via the air sampler method, or a simple impingement method, and calculating the colony-forming units per cubic meter of air (cfu/m^3^) [7, 8]. Many studies have reported on airborne microorganisms in hospitals [9–11]. Airborne bacteria and fungi may cause several adverse effects, especially infectious, allergenic, and immunotoxic disorders. Microbiological air quality is an important criterion that must be taken into account when indoor workplaces are designed to provide a safe environment. The present study provides information on the indoor air concentrations of microorganisms and focuses on the assessment of airborne bacterial and fungal communities in various wards of two hospitals (Kashani and Hajar) in Shahrekord city. It also investigates the effect of several factors, including type of hospital, location, time, type of microorganisms, and concentration. The aforementioned hospitals had 11, 9 wards, 340, 350 beds, 9, 4 floors, and 393, 476 staffs, respectively.

## 2. Methodology

### 2.1. Study Area

The current cross-sectional study evaluated two hospitals (Kashani and Hajar) of Charharmahal va Bakhtiari province in the southwest of Iran for three months.

### 2.2. Sample Collection and Analysis

**Figure 1:**
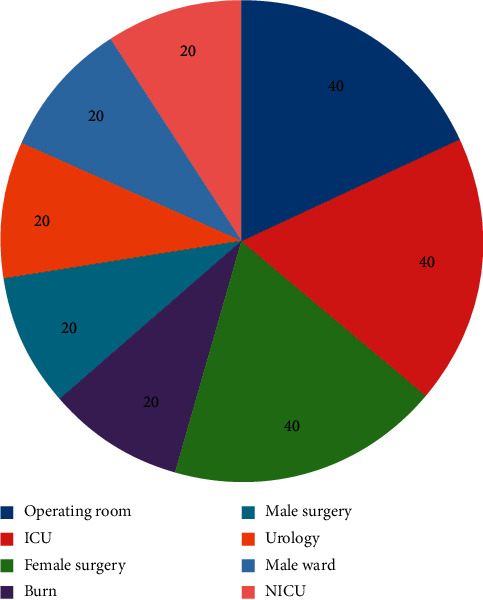
The frequency of samples based on hospital sites.

In Kashani hospital, 120 samples were collected from six selected wards: male surgery, female surgery, burns ward, urology, intensive care unit (ICU), and operating room. In Hajar hospital, 100 samples were collected from five selected wards: female surgery, male ward, infant critical care unit (NICU), ICU, and operating room. The air samples were collected twice every day, in the morning between 10am and 12pm, and in the afternoon between 3 and 5pm, three days per week. The sampling time was covered before, during, and after visiting hours, which are 2pm–4 pm.  Figure 1 shows the frequency of samples based on the hospitals sites. The temperature in the selected study sites ranged from 23°C to 28°C.

### 2.3. Air Sampling

Air sampling measures the viable enumeration of bacteria and fungi. We conducted air sampling in the two hospitals for 3 months to determine the indoor air quality (IAQ). Air sampling for the presence of culturable viable counts of bacteria and fungi was conducted at respiratory height (at 1.5 meters) for 5 minutes using Quick Take 30 Sample Pump (SKC, Eight Four, PA, USA) based on the principle of air impaction. The plates were exposed for about 1 hour at strategic locations (1 m above the floor, and 1 meter away from the wall) in the ward and transported in a clean container to the laboratory for microbiological examination immediately after collection. The pump was operating at a flow rate of 28.3 liters per minute (LPM) and sucked through polytetrafluoroethylene (PTFE) membranes (47 mm diameter, 0.4 *μ*m pore size). Total bacterial and fungal microorganisms were counted and reported as colony-forming units per cubic meter of air (cfu/m^3^) for impingement methods [12, 13]. For the sedimentation method, microorganisms were calculated as total cfu_s_ per plate per hour (cfu/plate/h) [14].

### 2.4. Analytical Media

Bacterial isolates were identified via Gram stain, growth on MacConkey and triple sugar iron agar, oxidase test, oxidation–fermentation, catalase test, and IMVIC reactions [15, 16]. Saboroud Dextrose Agar (SDA) was used to determine fungal counts. Fungal colonies were identified to the genus and species level via macroscopic and microscopic features [17]. The sampled plates were incubated at 35–37°C for 24–48 h in the case of bacterial analysis and at 22–24°C for 5-6 days in the case of fungal sampling. The culture media were provided according to the manufacturer and sterilized by autoclaving at 121°C for 15 minutes.

### 2.5. Data Analysis

Descriptive statistical parameters, such as the mean, standard deviation, median, minimum, and maximum, were applied to the data. *T*-test and Mann-Whitney test were used to determine the level of significance at *p* < 0.05.

## 3. Results

### 3.1. Location and Time of Sampling

The concentrations of airborne bacteria and fungi at the hospitals were different at different sites from 43.75 ± 20.32 to 243.75 ± 40.03 cfu/m^3^ (Table 1). The Mann-Whitney test showed that mean contamination was higher in Kahani hospital (132.33 ± 82.70) than in Hajar hospital (54.30 ± 15.62) (*p* < 0.05). The highest airborne bacteria and fungi exposure was found in the male surgery in Kashani hospital to be 243.75 ± 40.03 cfu/m^3^ (Table 1). *T*-test was used to compare similar wards (i.e., female surgery, operating room, and ICU) in both hospitals. *T*-test reveals the significant difference in the total contamination of operating rooms in Kashani and Hajar (*p* < 0.05) (Table 2). The paired *t*-test showed no significant difference between total contamination of ICU Kashani and Hajar (*p* > 0.05) (Table 2), and between total contamination of ward female surgery in Kashani and Hajar (*p* > 0.05) (Table 2).

**Figure 2:**
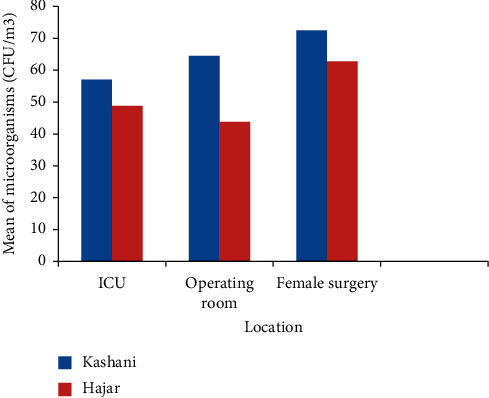
Comparison of mean microorganisms at similar wards in hospitals.

Comparison of mean contamination in Kashani hospital showed that the mean contamination in male surgery was greater than that in other wards, and in Hajar hospital the mean contamination in female surgery was greater than that in other wards.  Figure 2 shows a comparison of mean microorganisms at similar wards in hospitals. The mean of microorganisms Kashani hospital in the ICU, operating room, and female surgery ward were higher than those in Hajar hospital. The highest microorganism population was recorded in the afternoon (57%) between 3 pm and 5 pm compared to the morning (43%).

### 3.2. Bacteria

Table 3 indicates concentrations of microorganisms isolated from various wards in the hospitals. The highest bacteria contamination was found in the ICU ward in both hospital with 25%, and the maximum fungi contamination was found in the burns ward in Kashani hospital with 50% (Table 3).

Table 4 indicates the percentage and frequency of Gram-positive and Gram-negative bacteria isolates from various wards environments in the hospitals. Samples were Gram-negative (31.81%), Gram-positive (0.9%), and both genus bacteria (68.18%), respectively. The Gram-negative bacteria were *E. coli, Enterobacter, Salmonella, Serratia, Proteus, Klebsiella, Citrobacter,* and *Pseudomonas*. In the present study, the prevalent bacterial groups from indoor air at the general total included *Staphylococcus* spp*., Staphylococcus aureus, Staphylococcus saprophyticus, Staphylococcus epidermidis,* and *Staphylococcus lugdunensis.*

### 3.3. Fungi

The maximum fungal concentration was 280 (cfu/palate.h), while the minimum was 0 (cfu/palate.h) in all hospital wards (Table 5).

The highest airborne fungi concentration was in the burns ward in Kashani hospital of 280 cfu/plate/h (Tables 3 and 5). The fungal genera isolated included Yeast, *Aspergillus flavus, and Penicillium* (Table 6).

## 4. Discussion

The present study was performed at a preliminary level to evaluate the concentration of airborne microorganisms in various wards of two hospitals in Shahrekord, Iran. We investigated the microbial qualities of both hospitals using sedimentation (settle plate technique) and impingement methods. A total of 12 bacterial species were isolated and identified with varying frequencies of occurrence. The bacterial isolates *Staphylococcus* sp., *Escherichia coli*, *Salmonella*, *Enterobacter*, *Pseudomonas*, *Serratia Citrobacter*, *Proteus*, *Klebsiella*, *Staphylococcus aureus* (20.5%), and *Pseudomonas* (9.1%) were dominant among the genera identified in two hospitals of Shahrekord. This is similar to what was reported by Ekhaise and Ogboghodo who isolated 10 bacteria species from indoor air of two hospitals in Benin City, Nigeria [18]. Epidemiological studies have shown that high concentrations of microorganisms in the air can be allergenic; however, even low concentrations of some particular microorganisms can cause serious diseases [19]. According to the results of this research, the microorganisms isolated from hospitals, *Staphylococcus* sp.*, Salmonella, Pseudomonas, Enterobacter, and E. coli,* are known to be pathogenic. *E. coli* is a Gram-negative, facultatively anaerobic rod-shaped bacterium that is an indicator of recent fecal contamination. Most *E*. *coli* strains are harmless but some serotypes can cause serious food poisoning in humans and are occasionally responsible for food contamination. *S. aureus* is normally part of the skin flora. About 20% of the human populations are long-term carriers of *S. aureus* known to form aggregates in nature, so they tend to yield higher colony counts [20, 21]. One study has reported that the bacteria isolates from different wards and units of a teaching hospital in Nigeria were *Staphylococcus aureus*, *Klebsiella* sp., *Bacillus cereus*, *Bacillus subtilis*, *Streptococcus pyogenes*, and *Serratia marcescens* [22]. Another study reported the mean concentration for *Micrococcus*, *Viridians*, *Pneumococcus*, *Escherichia coli*, *Streptococcus*, *Bacillus cereus*, *B. subtilis*, *Staphylococcus aureus*, *Pseudomonas*, *Klebsiella*, *Citrobacter*, and *Enterobacter* to be 63.32 ± 32.94 cfu/m³ to 103.88 ± 33.84 [17].

The present study showed that the total number of airborne microbes was higher during the afternoon. A similar finding was reported in an Indian study in which the highest bacterial population (25 cfu/m^3^ to 725 cfu/m^3^) was recorded in the afternoon between 1 pm and 2 pm compared to the morning and evening [21]. The visitor population of the hospital environment increased during hospital visiting hours. In addition to the patients and staff, the visitors, their activities, and the items brought by them become additional sources of bioaerosols, and increased population activity in short term can suddenly increase the airborne bioaerosols level in hospitals [23–25]. Another study reported that *Pseudomonas, Bacillus* sp.*, Micrococcus* sp.*, Staphylococcus* sp.*, Exiguobacterium* sp.*, Sphingomonas* sp.*, Massilia* sp.*, Kocuria* sp.*, Fusarium* sp.*, and Aspergillus* sp. had colonized all the sampling sites within the hospital [20].

Different countries have different standards, so there is no similar international standard available on levels and acceptable maximum bioaerosol loads [26]. The study conducted by WHO on the evaluation of health risks of biological agents in indoor environments proposed that total microbial density must not exceed 1000 cfu/m^3^ [27, 28]. If higher than this, the environment is considered as contaminated [26–28]. Some studies considered that 300 cfu/m^3^ and 750 cfu/m^3^ should be the limit for fungi and bacteria, respectively [29, 30]. However, another study has suggested limits of 100 cfu/m^3^ for bacteria and 50 cfu/m^3^ for fungi in the hospital air [17].

The most common species were yeast (22.7%) and *Penicillium* (20.5%). Most fungi are known to be associated with asthma in both children and adults. These fungi lead to pulmonary aspergillosis when inhaled [31].

Most cases of contamination occur in people with underlying illnesses and low immunity level [32]. Approximately 49.1% of *Aspergillus* sp. outbreak within hospitals can be attributed to the construction work in or around hospitals [33]. [33]. Fungi isolated in the present study are identified as highly pathogenic or opportunistic pathogens/medically important microorganisms. One research reported the fungal level to range between 1 and 32 cfu/m^3^. The prevalent genera were *Penicillium* (41%) and *Aspergillus* (24%) [12]. These results are in agreement with those obtained by some studies on the average concentration of airborne microorganisms. One study reported that fungal concentrations ranged within 11–566 cfu/m^3^ in indoor air in hospitals [6]. The maximum and the minimum fungal concentration were 400 cfu/m^3^ and 105 cfu/m^3^, respectively. In a study conducted in Benin City, Nigeria, the fungal isolates of the hospitals indoor air included *Penicillium*, *Mucor*, *Aspergillus,* and *Fusarium* [18]. Airborne fungi and their spores have the potential to be blown into buildings with natural ventilation. They can pose a threat to the life of immunocompromised patients when are blown in through the windows of the relevant wards [34]. Another research found that the predominant pathogens in hospitals of Sari, Iran, include *Mucorales* sp.*, Candida* sp.*, Fusarium* sp.*, and Aspergillus* sp. [35]. One study conducted in Sri Lanka showed that *Aspergillus* sp., acquired by inhalation of airborne dust particles, causes aspergillosis infection, which is followed by pneumonia development, and the fungus disseminates through the bloodstream to other organs [20]. In the present research, operating room and ICU showed the lowest microbial counts than other wards; however, according to some other studies, the operating theatre and ICU showed the lowest microbial counts in comparison to other wards. The reasons for lower microbial concentration in the operating room and ICU were the improved conditions and good air conditioning methods as they are critical regions in the hospital [12, 21, 25].

The average levels of bacteria (1,414 cfu/m^3^) and fungi (290 cfu/m^3^) indicated that all hospital rooms were generally contaminated [7]. Results of our study showed that Kashani hospital had higher concentrations of bacterial and fungal organisms than Hajar hospital because it had a higher number of floors, rooms, staff, beds, and visitors.

## 5. Conclusion

Microorganisms mean concentration was about 43.75 ± 20.32 to 243.75 ± 40.03 cfu/m^3^. The most predominant bacterial and fungal isolates were *Staphylococcus aureus* (20.50%) and Yeast (22.70%). Kashani hospital had higher concentrations of bacterial and fungal organisms than Hajar hospital.

The prevalence of pathogenic organisms in this study indicates the lower degree of cleanliness in hospitals and, as such, poses a potential threat to the health and wellbeing of the patients and staff. Therefore, regular inspection of the bioaerosols concentration in hospital environment is recommended to improve the general wellbeing of patients. Generally, all the studied wards were contaminated with bacteria and fungi. The obtained bacteria and fungi concentrations of air might be potential risk factors for the spread of nosocomial infection in hospitals.

## Figures and Tables

**Table 1:** Mean concentrations of airborne microorganisms (bacteria and fungi) at various wards in the hospitals.

Hospital	Ward	Mean ± SD (CFU/m^3^ of air)^*∗∗*^
^*∗*^ *Kashani mean* *±* *SD* *=* *132.33* *±* *82.70*	Female surgery	72.5 ± 20.03
	Male surgery	243.75 ± 40.03
	Burns ward	200 ± 30.32
	Urology	133.75 ± 30.32
	Intensive care unit (ICU)	57 ± 19.32
	Operating room	64.5 ± 6.75

^*∗*^ *Hajar mean* *±* *SD* *=* *54.30* *±* *15.62*	Female surgery	62.75 ± 9.33
	Intensive care unit (ICU)	48.75 ± 21.27
	Operating room	43.75 ± 20.32
	Male ward	51.25 ± 17.27
	Infant critical care unit (NICU)	57.50 ± 22.27

^*∗*^A level of significance at *p* < 0.05. ^*∗∗*^Collected by impingement method.

**Table 2:** Mean concentrations of airborne microorganisms (bacteria and fungi) at similar wards in the hospitals.

Hospital	Ward	Sample	Mean ± SD (CFU/m^3^ of air)^*∗*^	Std error mean
Kashani	Operating room	*N* = 20	64.5 ± 6.75	1.50
Hajar		*N* = 20	43.75 ± 20.32	4.55

Kashani	ICU	*N* = 20	57 ± 19.32	4.32
Hajar		*N* = 20	48.75 ± 21.27	4.75

Kashani	Female surgery	*N* = 20	72.5 ± 20.03	4.48
Hajar		*N* = 20	62.75 ± 9.33	2.08

^*∗*^A level of significance at *p* < 0.05.

**Table 3:** Concentrations of microorganisms isolated from various wards in the hospitals.

Type of ward	Frequency and type of microorganism			Percentage and type of microorganism		
	Bacteria	Fungi	Bacteria and fungi	Bacteria	Fungi	Bacteria and fungi
Operating room	5	0	35	12.5	0	87.5
ICU	10	0	30	25	0	75
Female surgery	5	0	35	12.5	0	87.5
Burns ward	0	10	10	0	50	50
Male surgery	0	0	20	0	0	100
Urology	0	0	20	0	0	100
Male ward	0	0	20	0	0	100
NICU	0	0	20	0	0	100
Total	20	10	190	9.1	4.54	86.36

**Table 4:** Percentage and frequency of Gram-positive and Gram-negative bacteria isolated from various wards in the hospitals.

Type of bacteria	Frequency	Percentage
No	10	4.5
*Staphylococcus aureus*	45	20.5
*Staphylococcus epidermidis*	5	2.3
*Enterobacter*	5	2.3
*Pseudomonas*	20	9.1
*Salmonella*	10	4.5
*Escherichia coli*	5	2.3
*Enterobacter, Staphylococcus aureus, Staphylococcus epidermidis*	10	4.5
*Staphylococcus aureus, St. lugdunensis, Klebsiella, Serratia*	5	2.3
*Staphylococcus aureus, Serratia*	5	2.3
*Staphylococcus aureus, St. saprophyticus*	5	2.3
*Salmonella, Serratia*	10	4.5
*Staphylococcus aureus, St. saprophyticus, Pseudomonas*	5	2, 3
*Staphylococcus aureus, Pseudomonas*	10	4.5
*Staphylococcus aureus, St. lugdunensis, Serratia*	5	2.3
*Staphylococcus aureus, St. lugdunensis, St. lugdunensis, Salmonella*	5	2.3
*Staphylococcus lugdunensis, Salmonella, Salmonella*	5	2.3
*Staphylococcus aureus, St. lugdunensis, Salmonella*	5	2, 3
*Staphylococcus lugdunensis, Citrobacter, Proteus*	10	4.5
*Staphylococcus epidermidis, Enterobacter*	5	2.3
*Staphylococcus lugdunensis, Citrobacter, Klebsiella*	5	2.3
*Staphylococcus aureus, Klebsiella*	5	2.3
*Staphylococcus aureus, St. lugdunensis*	5	2.3
*Staphylococcus aureus, Citrobacter, Escherichia coli*	5	2.3
*Staphylococcus lugdunensis, Pseudomonas,*	5	2.3
*Staphylococcus aureus, Salmonella*	5	2.3
*Staphylococcus aureus, St. Lugdunensis, Salmonella, Escherichia coli*	5	2.3
Total	220	100

**Table 5:** Concentrations of fungi in the various wards in the hospitals.

Concentrations of fungi (cfu/palate.h)^*∗*^	Frequency	Percentage
0	20	9.1
25	60	27.3
30	20	9.1
35	20	9.1
40	20	9.1
45	10	4.5
50	10	4.5
70	5	2.3
80	5	2.3
90	5	2.3
120	5	2.3
150	10	4.5
200	5	2.3
215	5	2.3
240	5	2.3
250	10	4.5
280	5	2.3
Total	220	100

^*∗*^Collected by sedimentation method.

**Table 6:** Airborne fungi isolated from various wards in the hospitals.

Type of fungi	Frequency	Percentage
No	20	9.1
Yeast	50	22.7
*Penicillium*	45	20.5
*Penicillium*, *Aspergillus*	15	6.8
*Aspergillus*, yeast	10	4.5
*Penicillium*, yeast	35	15.9
*Penicillium, Aspergillus*, yeast	45	20.5
Total	220	100

## Data Availability

The data used to support the findings of this study are included within the article.

## References

[1] Degois J., Clerc F., Simon X., Bontemps C., Leblond P., Duquenne P. (2017). First metagenomic survey of the microbial diversity in bioaerosols emitted in waste sorting plants. *Annals of Work Exposures and Health*.

[2] Małecka-Adamowicz M., Kubera Ł., Jankowiak E., Dembowska E. (2019). Microbial diversity of bioaerosol inside sports facilities and antibiotic resistance of isolated Staphylococcus spp. *Aerobiologia*.

[3] Yoo K., Lee T. K., Choi E. J. (2017). Molecular approaches for the detection and monitoring of microbial communities in bioaerosols: a review. *Journal of Environmental Sciences*.

[4] Reanprayoon P., Yoonaiwong W. (2012). Airborne concentrations of bacteria and fungi in Thailand border market. *Aerobiologia*.

[5] Mandal J., Brandl H., Bioaerosols (2011). Indoor environment-a review with special reference to residential and occupational locations. *The Open Environmental & Biological Monitoring Journal*.

[6] Osman M., Ibrahim H., Yousef F., Elnasr A. A., Saeed Y., Hameed A. A. (2018). A study on microbiological contamination on air quality in hospitals in Egypt. *Indoor and Built Environment*.

[7] Ilić P., Božić J., Ilić S. (2018). Microbiological air contamination in hospital. *International Journal of Progressive Sciences and Technologies (IJPSAT)*.

[8] Tong X., Xu H., Zou L. (2017). High diversity of airborne fungi in the hospital environment as revealed by meta-sequencing-based microbiome analysis. *Scientific Reports*.

[9] Kim K. Y., Kim Y. S., Kim D. (2010). Distribution characteristics of airborne bacteria and fungi in the general hospitals of Korea. *Industrial Health*.

[10] Sautour M., Sixt N., Dalle F. (2009). Profiles and seasonal distribution of airborne fungi in indoor and outdoor environments at a French hospital. *Science of the Total Environment*.

[11] Karalti I., Çolakoğlu G. (2012). The seasonal distribution of airborne fungi in two hospitals in Istanbul. *African Journal of Biotechnology*.

[12] Verde S. C., Almeida S. M., Matos J. (2015). Microbiological assessment of indoor air quality at different hospital sites. *Research in Microbiology*.

[13] Pereira L. D., Raimondo D., Corgnati S. P., da Silva M. G. (2014). Assessment of indoor air quality and thermal comfort in Portuguese secondary classrooms: methodology and results. *Building and Environment*.

[14] Mandin C., Trantallidi M., Cattaneo A. (2017). Assessment of indoor air quality in office buildings across Europe - the OFFICAIR study. *Science of the Total Environment*.

[15] Park D.-U., Yeom J.-K., Lee W., Lee K.-M. (2013). Assessment of the levels of airborne bacteria, gram-negative bacteria, and fungi in hospital lobbies. *International Journal of Environmental Research and Public Health*.

[16] Donguk P., Jeong-Kwan Y., Won Jae L., Kyeong-Min L. Assessment of the levels of airborne bacteria, gram negative bacteria, and fungi in hospital lobbies.

[17] Mirzaei R., Shahriary E., Qureshi M. I., Rakhshkhorshid A., Khammary A., Mohammadi M. (2014). Quantitative and qualitative evaluation of bio-aerosols in surgery rooms and emergency department of an educational hospital. *Jundishapur Journal of Microbiology*.

[18] Ekhaise F. O., Ogboghodo B. (2011). Microbiological indoor and outdoor air quality of two major hospitals in Benin City, Nigeria. *Sierra Leone Journal of Biomedical Research*.

[19] Basińska M., Michałkiewicz M., Ratajczak K. (2019). Impact of physical and microbiological parameters on proper indoor air quality in nursery. *Environment International*.

[20] Sivagnanasundaram P., Amarasekara R., Madegedara R., Ekanayake A., Magana-Arachchi D. (2019). Assessment of airborne bacterial and fungal communities in selected areas of teaching hospital, kandy, Sri Lanka. *BioMed Research International*.

[21] Bhatia L., Vishwakarma R. (2010). Hospital indoor airborne microflora in private and government-owned hospitals in Sagar City, India. *World Journal of Medical Sciences*.

[22] Awosika S., Olajubu F., Amusa N. (2012). Microbiological assessment of indoor air of a teaching hospital in Nigeria. *Asian Pacific Journal of Tropical Biomedicine*.

[23] Setlhare G., Malebo N., Shale K., Lues R. (2014). Identification of airborne microbiota in selected areas in a health-care setting in South Africa. *BMC Microbiology*.

[24] Nunes Z. G., Martins A. S., Altoe A. L. F. (2005). Indoor air microbiological evaluation of offices, hospitals, industries, and shopping centers. *Memórias Do Instituto Oswaldo Cruz*.

[25] Qudiesat K., Abu-Elteen K., Elkarmi A., Hamad M., Abussaud M. (2009). Assessment of airborne pathogens in healthcare settings. *African Journal of Microbiology Research*.

[26] Fekadu S., Getachewu B. (2015). Microbiological assessment of indoor air of Teaching hospital wards: a case of Jimma University specialized hospital. *Ethiopian Journal of Health Sciences*.

[27] World Health Organization (1988). WHO regional publications european series, No. 31: indoor air quality: biological contaminants.

[28] Hospodsky D., Qian J., Nazaroff W. W. (2012). Human occupancy as a source of indoor airborne bacteria. *PLoS One*.

[29] Paramita S., Yadi Y. (2017). Microbiological assessment of indoor air of takalar county hospital wards in south sulawesi, Indonesia. *Science Journal of Public Health*.

[30] Fusarium T. (2016). Microbiological assessment of indoor air quality at different sites of a tertiary hospital in South-South Nigeria. *Port Harcourt Medical Journal*.

[31] Jaakkola J. J. K., Hwang B.-F., Jaakkola M. S. (2010). Home dampness and molds as determinants of allergic rhinitis in childhood: a 6-year, population-based cohort study. *American Journal of Epidemiology*.

[32] Shiaka G., Yakubu S. (2013). Comparative analysis of airborne microbial concentrations in the indoor environment of two selected clinical laboratories. *IOSR Journal of Pharmacy and Biological Sciences (IOSR-JPBS)*.

[33] Vonberg R. P., Gastmeier P. (2006). Nosocomial aspergillosis in outbreak settings. *Journal of Hospital Infection*.

[34] Tang J. W., Nicolle A., Pantelic J. (2013). Different types of door-opening motions as contributing factors to containment failures in hospital isolation rooms. *PLoS One*.

[35] Moazeni M., Asgari S., Nabili M. (2018). Nosocomial fungal infections: epidemiology, diagnosis, treatment and prevention. *Journal of Mazandaran University of Medical Sciences*.

